# Motivational Interviewing: moving from why to how with autonomy support

**DOI:** 10.1186/1479-5868-9-19

**Published:** 2012-03-02

**Authors:** Ken Resnicow, Fiona McMaster

**Affiliations:** 1 University of Michigan, School of Public Health, Department of Health Behavior and Health Education, 109 Observatory Street, Room 3867 SPH I, Ann Arbor, MI 48109-2029, USA

## Abstract

Motivational Interviewing (MI), a counseling style initially used to treat addictions, increasingly has been used in health care and public health settings. This manuscript provides an overview of MI, including its theoretical origins and core clinical strategies. We also address similarities and differences with Self-Determination Theory. MI has been defined as *person-centered method of guiding to elicit and strengthen personal motivation for change*. Core clinical strategies include, e.g., reflective listening and eliciting change talk. MI encourages individuals to work through their ambivalence about behavior change and to explore discrepancy between their current behavior and broader life goals and values. A key challenge for MI practitioners is deciding when and how to transition from building motivation to the goal setting and planning phases of counseling. To address this, we present a new three-phase model that provides a framework for moving from WHY to HOW; from building motivation to more action oriented counseling, within a patient centered framework.

## Introduction

Motivational Interviewing (MI) is a counseling style initially used to treat addictions [1-5]. Its efficacy has been demonstrated in numerous randomized trials across a range of conditions and settings [5-8]. Over the past 15 years, there have been considerable efforts to adapt and test MI across various chronic disease behaviors [7,9-21]. This article provides an overview of MI and its philosophic orientation and essential strategies, with an emphasis on its application to health promotion and chronic disease prevention. Because many practitioners find it difficult deciding when and how to transition from building motivation to the goal setting and planning phases of counseling, we present a new three-phase model that provides a framework for helping clinicians transition from the WHY to HOW phase; from building motivation to more action oriented counseling. Further, we discuss possible connections between elements of the three phase MI model and Self-Determination Theory.

### Overview of Motivational Interviewing

MI is an egalitarian, empathetic "way of being". It is a communication style that uses specific techniques and strategies such as reflective listening, shared decision-making, and eliciting change talk. Recently it has been defined as a "person-centered method of guiding to elicit and strengthen personal motivation for change*" *[22]. An effective MI practitioner is able to strategically balance the need to "comfort the afflicted" and "afflict the comfortable"; to balance the expression of empathy with the need to build sufficient discrepancy to stimulate change.

One goal of MI is to assist individuals to work through their ambivalence or resistance about behavior change. MI appears to be particularly effective for individuals who are initially resistance to change [3,20,23-25]. Conversely with highly motivated individuals it may be counterproductive[26]. The tone of MI is nonjudgmental and encouraging. Counselors establish a non-confrontational and supportive climate in which clients feel comfortable expressing both the positive and negative aspects of their current behavior. Ambivalence is explored prior to moving toward change.

Although MI is client-centered, unlike classic Rogerian therapy, it is more goal-driven and directional. That is, there is a clear positive behavioral outcome, e.g., quitting smoking, losing weight, adhering to medication. Given this directionality, some MI practitioners feel it is important to make explicit their "bias" in this regard. However, to maintain client autonomy they may also explicitly communicate that any decision is ultimately up to client. When MI is used to discuss preference sensitive decisions such as obtaining a genetic test or choosing breast conserving vs. mastectomy for breast cancer treatment, some MI theoreticians contend that in these situations the exchange is no longer MI, because there is not a clear behavioral goal. Conversely, in such situations the goal could be conceptualized as making a shared and informed decision, which then could be considered a directional outcome, and still within the rubric of MI.

Whereas many counseling models rely heavily on therapist insight and directive advice, in MI patients themselves do much of the psychological work. They generate the rationale for change. Unlike cognitive-behavioral interventions [22], MI counselors generally make no direct attempt to dismantle denial or confront irrational or maladaptive beliefs. Instead they may subtly help clients detect possible contradictions in their thoughts and actions; to experience discrepancy between their current actions and who they ideally want to be. MI counselors rarely attempt to convince or persuade. Instead, the counselor subtly guides the client to think about and verbally express their own reasons for and against change and explore how their current behavior or health status may impact their ability to achieve their life goals or align with their core values. MI encourages clients to make fully informed and deeply contemplated life choices, even if the decision is not to change.

### Theoretical Underpinnings

MI arose from intuitive clinical practice rather than any particular theoretical model [5]. (see Vansteenkiste et al and Patrick & Williams in this issue). It emerged in part as an alternative to the directive and even confrontational style of substance use counseling commonly used throughout the 1980s[5]. Many of its principals and techniques are rooted in the client-centered approach of Rogers and Carkauff, although MI is perhaps more goal driven (and unidirectional) than classic Rogerian client-centered therapy [22,27-29]. Despite MI's largely atheoretical origins, in recent years, an increasing number of MI researchers and practitioners have begun to use self-determination theory (SDT) as a de facto model for understanding how and why MI works [30,31]. Originally proposed by Deci and Ryan [30,32,33], SDT conceptualizes a continuum of human motivational regulation [34-36], ranging from amotivated to fully intrinsic, and allows that humans can experience multiple types of motivational regulations simultaneously.

*External *regulation, one type of controlled regulation, includes extrinsic rewards and punishments administered by other people. It includes, in addition to financial and legal constraints, pressure from other people for the person to change, often in the form of social sanction. Whereas external regulation may temporarily motivate change, such change is seen as less enduring and less stable, particularly if more autonomous forms of self-regulation are low. Another form of controlled regulation is *introjected regulation*, whereby a person is motivated not by external controls, but by internalized self-judgment. Introjections involve some degree of negative self-reference such as shame, guilt, or social comparison, and in this sense they are seen as 'self-controlling'. Clients often come into counseling with higher levels of those two types of controlled motivation, or being *amotivated (without intention to change, often feeling unable to change)*. A key challenge for the MI practitioner is to help the client become more autonomously motivated [30,31], as these forms of self-regulation are associated with long term change (see Patrick and Williams in this issue). *Identification *is the first type of the more self-determined (or autonomous) form of regulation. It conveys a sense that change is personally important and meaningful. The most autonomous form of extrinsic motivation is *integrated regulation*. Here the person not only sees the importance of change, but also links the change with his or her other core values and beliefs. Change arising from integrated regulation is seen as the most stable and enduring. The person finds meaning in his or her "suffering." Fully *intrinsically motivated behaviors *are novel or satisfying in their own right. They are engaged in for inherent enjoyment rather than any symbolic value. They do not need any symbolic or constructed motivation. This type of motivation, however, is less common as a reason to initiate most behavior change, because most health promoting behaviors are often not perceived as inherently more enjoyable than the riskier behaviors they might be replacing and are perhaps therefore, not intrinsically motivating[34-36]. However, some aspects of positive behavior change, e.g. engaging in physical activity or the successful stopping of an addictive behavior, can provide feelings of novelty and challenge and therefore could be seen as intrinsically motivating.

SDT also proposes three fundamental human needs that are relevant for motivating behavior change: Competence, relatedness, and autonomy [30,31]. All three needs are consistent with the philosophy and delivery of MI. Competence, akin to the concept of self-efficacy in social cognitive theory [37], describes people's confidence in their ability to execute change. Building efficacy for change is a core concept of MI, as reflected by MI practitioners' widespread use by of the 0-to-10 confidence ruler. Competence support can also manifest as "pulling forward" successes from prior behavior change attempts or building a change plan with realistic goals that build efficacy and encourage persistence. Relatedness involves the need for meaningful social connection, which is often integrated into MI through the use of the values clarification activity and through the relationship established with an empathetic, nonjudgmental counselor. Finally, autonomy in SDT is related to people's need to feel volitional in their actions rather than feeling controlled.

Autonomy support is central to the practice of MI[30]. It is promoted through strategies such as eliciting and acknowledging (or reflecting) client perspectives and values, shared agenda setting, providing a menu of effective choices for what is discussed and what goals are set, and an overall lack of coercion and direct persuasion throughout the encounter. MI also promotes autonomous behavior change by linking change to the person's broader goals, values, and sense of self. Measures of controlled and autonomous motivation drawn from SDT have been shown to have a mediating role in MI interventions[38]. G. Williams has developed a counseling approach, Autonomy Supportive Therapy, which although directly rooted in SDT shares many MI principles[33,39,40]. (For more on this, see Patrick and Williams in this issue).

Despite the many similarities between the theory of SDT and the practice of MI, subtle, although potentially important differences have been identified. For example, whereas MI emphasizes the amount, intensity and sequence of change talk as essential elements of the change process[5,41], SDT might place greater emphasis on the quality of the change talk [42]. Specifically, SDT would hypothesize that integrated and identified change talk should produce more sustained change than talk that has an introjected tone or reflects external pressure, even if such expressions are strong in intensity (See also Vansteenkiste et al in this issue). On the other hand, MI perhaps places greater emphasis on the source of autonomy, that is, effort is placed to ensure that motivation, solutions, and action plans emanate from the client, whereas from an SDT perspective, it may be more important to ensure client volition, even if the initial source of motivation and advice is external (i.e., from the clinician) [30,42], as long as that advice is nonjudgmental (e.g., advice given regarding what behaviors and treatments are likely to improve well being, and not given to control the patients behavior) and it includes the option of not changing the behavior. In this special circumstance of SDT in medical care, advice delivered in a need supportive manner is predicted to be internalized as autonomous self-regulation over time.

Another difference lies in how SDT conceptualizes ambivalence. In MI, clients are often assumed to possess both strong reasons for and against change. Given that the SDT continuum implies motivation is discrete, it is not clear how simultaneous motivations are addressed. Similarly it is not clear how resistance is conceptualized within SDT. Clients expressing resistance would most likely be viewed as experiencing controlled regulation, either through external pressure or introjected motivation. The goal then, from an SDT perspective would be to help the client find more autonomous reasons for change, which should soften resistance. In MI however, rolling with resistance is seen as a core strategy for softening resistance. Perhaps SDT might consider such reflections autonomy supportive as they accept the person as they are and do not try to push the person to think differently.

MI has also been linked to complexity science and chaos theory [43,44]. Resnicow et al have suggested that motivation to change one's behavior can be viewed as a perfect storm of intrapsychologic events--a complex, nonlinear interplay of thoughts and feelings that compel the person to change[43,44]. Motivation is not seen as the gradual or intellectual process of decisional balance but a much more discrete event; an epiphany. Such "sudden gains" in motivation have been observed in smoking cessation [45,46] and the treatment of depression [47-49]. To achieve such "quantum change", MI practitioners provide clients with an opportunity to consider their life with and without their risk behaviors and to explore how change can propel them forward in life. This process can lead to a motivational epiphany, whereby the client feels a compelling reason to change that was not heretofore present[43,44]. The transformation is difficult to predict in part because the system is sensitive to initial conditions, i.e., small differences in the starting point can create large changes in outcomes. Motivation can be dramatically altered by small inputs.

### Key MI Strategies

The essence of MI lies in its spirit; however, specific techniques and strategies, when used effectively, help ensure such spirit is evoked. To this end, MI counselors rely heavily on reflective listening, rolling with resistance, and eliciting change talk.

Reflective listening, a core component of client-centered counseling, can be conceptualized as a form of hypothesis testing. The hypothesis can be stated in generic terms as "If I heard you correctly, this is what I think you are saying..." or "Given what you said, you might feel xxxx....." Reflections, particularly by counselors who are new to the technique, often begin with the phrase, "It sounds like...." More skilled counselors often phrase their reflections in a more truncated form, such as "You are having trouble with...", leaving off the assumed "It sounds like...." The goals of reflecting include demonstrating that the counselor has heard and is trying to understand the client, affirming the client's thoughts and feelings without judgment, and helping the client continue the process of self-discovery. Even when reflections are inaccurate, through the act of correcting the counselor, clients may clarify their thoughts and feelings and move the discussion forward. This is sometimes referred to as a productive miss or a "foul tip."

One of the most important elements of mastering MI is suppressing the instinct to respond with questions or premature advice. Questions can be biased by what the counselor may be interested in hearing about, their worldview or prior experience, rather what the client wants or needs to explore. Premature advice, in turn, can elicit resistance or pseudo-commitment. Reflecting helps ensure that the direction of the encounter remains client-driven. The simplest level of reflection tests whether the counselor understood the content of the client's statement. Deeper levels explore the meaning or feeling behind what was said. Effective deeper-level reflections can be thought of as the next sentence or next paragraph in the story, i.e., "where the client is going with it." Reflections involve several levels of complexity or depth [28]. We describe seven types of reflections, the final two, reflections on omission and action reflections, are new variants.

#### Reflections

##### Content reflections

Content reflections are used to elicit the basic facts in the client's story. Although it is perhaps the simplest and least powerful type of reflection, content reflections can be important when trying to gather background information and build initial rapport. They generally entail paraphrasing what the client just said but without adding much to the client's initial statement. To avoid parroting, the counselor still slightly changes the client's words. These reflections generally require less risk and less inference than the other types.

##### Feeling/meaning reflections

Feeling/meaning reflections often take the form of "You are feeling xxx about xxx or because of xxx." Meaning reflections may also include a statement about why the person feels a certain way, the symbolic meaning of the event or emotion, or how a feeling or action may be related to other important aspects of the person's life. Often practitioners are reluctant to use emotionally intense words. Glossing over or minimizing client feelings can communicate counselor discomfort with emotional intensity and shut the client down. Conversely, acknowledging emotional intensity is a powerful way to quickly build rapport and encourage the client to fully disclose their thoughts and feelings.

#### Rolling with resistance

Confronting clients can evoke reactance and shut them down[2]. Therefore, MI counselors "roll with resistance" rather than attempt to argue with the client. Such reflections can be thought of as "comforting the afflicted." The counselor "pull up alongside clients," essentially agreeing with the client, even if the statement is factually incorrect or unfairly places blame on others. Examples include: "You really enjoy smoking weed. You look forward to lighting up at night, and giving it up seems very difficult" or "eating at McDonalds is a real treat for you. It's cheap, convenient, and really works given your busy schedule". Such reflections help capture the client's reasons for not changing and allow them to express their resistance without feeling pressured to change or worrying about being judged.

##### Amplified negative reflections

Sometimes rolling with resistance is not sufficient to move the client forward. When this occurs an amplified negative reflection, that "afflicts the comfortable" may be appropriate. Paradoxically, amplified negative reflections are a way of arguing against change by exaggerating the benefits of or minimizing the harm associated with a risky behavior. It may take the form of "so you see no benefit in changing XX" or "XX is all positive for you." The counselor, by arguing against change can exhaust the client's negativity. In response, clients will often then reverse their course, and start to argue for change. This type of reflection poses some potential risks, and can occasionally backfire. Important here, is for the counselor to avoid any tone of sarcasm. This type of reflection is particularly useful when clients appear stuck in a "yes, but" mindset.

##### Double-sided reflections

capture client ambivalence and communicate to the client that the counselor heard their reasons both for and against change; that the counselor understands the decision is complex, and they are not going to prematurely push them to change. Double-sided reflections typically take the form of "on the one hand, you would like to change XX, but on the other hand changing XX would mean giving up XX" or "you are torn about changing XX...."

##### Reflection on omission

Sometimes a counselor can reflect back to clients what they have *not *said. This can include reflecting on the client's silence or reluctance to talk about a particular issue; "you don't seem like talking today or you didn't have much of a reaction to what I just said. " In such cases, an omission reflection can be seen as an extension of rolling with resistance. However, an additional permutation includes reflecting back to the client beliefs, solutions to problems, sources of help, etc that have not been raised. For example, if an otherwise happily married woman states that she has no one to exercise with, the counselor could reflect back "so it sounds like your husband is not the answer." Another variation might include, "so I assume you probably have thought about trying XX solution/option but that doesn't seem to work for you."

##### Action reflections

are a key tool in the guiding and choosing phases described later. They incorporate into the reflection, possible solutions to the client's barriers or a potential course of action. They can be essential in establishing specific action steps for change, in an autonomy supportive rather than prescriptive style. Action reflections may be seen as the bridge between HOW and WHY styles of counseling. They differ from the more common type of reflections such as those that focus on client feelings, rolling with resistance, or acknowledging ambivalence as they usually contain a potential concrete step that the client has directly or obliquely mentioned. The action reflection looks forward rather than inward or backward [5,41,50,51]. Because the client directly mentioned or alluded to the possible course (s) of action contained within the action reflection or they flow logically from the parameters established by the client, this type of reflection should not be confused with unsolicited advice. Like any type of reflection, ARs represent the clinician's best guess for what the client has said or more apropos here, where the conversation might be heading.

Action reflections can include multiple choices to support the client's autonomy. For example; "based on what you said there seems to be several possible options including × and Y". Because the client directly mentioned or alluded to these possible courses action, this type of reflection should not be confused with unsolicited advice (something generally discouraged in MI). There are four subtypes of action reflections:

##### Invert Barrier

This is the simplest and often the default type. It generically takes the form of "Sounds like, in order to move forward, you might want to address barriers a, b, and c." Specifically, in the case of smoking cessation this could entail, "so coming up with ways to address the cravings you experienced last time might help make quitting easier." Or, for weight control: "finding something in the morning that satisfies your sweet tooth but is a better choice than a donut might be useful" In this form, a discrete solution is not included in the reflection, but encourages the client to generates specific options, appropriate for their unique circumstances.

##### General Behavior Fix

Here, the action is presented in a non-specific way as more an umbrella strategy, with the intent of having the client fill in the details, for example, "So you might consider doing something like x, y, or z." Specifically, for smoking cessation counseling this could entail, "so a medicine to help reduce cravings might help make quitting easier." For obesity counseling a variant could be; "so something sweet like fruit or slightly sweetened cereal might help satisfy your sweet tooth in the morning." Importantly in both cases the client would have at least mentioned previously some desire to find a way to handle withdrawal if they were to quit smoking or in the case of the overweight patient, they would have mentioned that they enjoy having sweet versus savory food in the morning and that they like fruit and/or cereal. An advantage of the general fix is that the client can generate the specific strategy which can increase commitment and autonomy.

##### Specific Behavior Fix

Whereas the general fix may not include a discrete action step, sometimes based on prior discussion, there is a clear solution (or multiple solutions) that the client has mentioned or alluded to that well match their needs. In these cases, a more specified reflection may be effective. For example, "Sounds like doing × may be a possibility." Specifically, this could entail, "so a medicine like nicotine replacement, Zyban^©^, or Chantix^© ^that help reduce cravings might help make quitting easier." For weight control; "given you like bananas and yogurt, that might be an option for you instead of a chocolate croissant"

##### Cognitive Fix

Whereas the reflections above focus on behavioral actions, sometimes moving forward can entail modifying cognitions. This technique allows the implementation of cognitive therapy strategies within an MI framework. These reflections can be similar to cognitive restructuring techniques, although the new or alternative cognition is presented in the form of a reflection rather than directive advice.

A generic form would be, "sounds like in order to move forward, you may have to think about × differently". Common cognitive changes can include not applying all-or-nothing thinking, making peace with lack of immediate benefit or even short term discomfort, and understanding from prior experiences that they will be able to endure the discomfort

or they have been successful in similar prior situations. Another variant is helping the client view their effort, even if not resulting in success, as a positive expression of commitment rather than a failure of execution. Specifically, this could entail, "so not addressing this is an all or nothing thing or accepting the fact that you have dealt with similar discomfort in the past might help make quitting easier."

Suggesting new ways of thinking could also include reflections that help the client accept that that even small changes can be viewed as success (e.g., cutting down on drinking or smoking is a significant step forward) or using the example of physical activity, that incidental activity still "counts" toward ones physical activity. For example; "it might be useful for you to consider all your activity when you calculate your daily goals" or "you seem to only include your time in the gym as physical activity but not your walking to and from work or your gardening". Another variant is helping the client view their efforts, even if not resulting in success, as a positive expression of commitment rather than a failure.

#### Change Talk

A core principle of MI is that individuals are more likely to accept and act upon opinions that they voice themselves [52]. The more a person argues for a position, the greater his or her commitment to it often becomes. Therefore, clients are encouraged to express their own reasons and plans for change (or lack thereof). This process is referred to as eliciting change talk. Expression of change talk, particularly a strong crescendo of commitment, appears to be a good predictor of future change, and a key mediator of the MI process[5,53]. One commonly used technique to elicit change talk uses the importance/confidence rulers [23,54,55]. This strategy typically begins with two questions: (1) "On a scale from zero to ten, with ten being the highest, how important is it to you to change [insert target behavior]?" and 2) "On a scale from zero to ten, with ten being the highest and assuming you want to change this behavior, how confident are you that you could (insert target behavior)?" These two questions assess the importance that clients attribute to change and their confidence in being able change, respectively [1,54]. Clinicians typically follow each of these questions with two probes. If the client answered "five," for example, the counselor would probe first with, "Why did you not choose a lower number, like a three or a four?", followed by "What might it take to get you to a higher number, like a six or a seven?" These probes elicit positive change talk and ideas for potential solutions from the client. Other related questions that can be useful in determining motivation include, "how much energy do you feel it would take to change XX", "how much do you dread giving up XX?", and "how hopeful are you that you are going to be able to XX?". The latter is often reserved for the end of Phase II, after an action plan has been determined.

A related strategy is to help clients experience discrepancy between their current behavior and their personal core values or life goals; this can lead to values clarification and "afflicting the comfortable." Clients choose from a list of values (see Table 1) three to five that are important to them. The counselor next asks how if at all the client might connect the health behavior in question with his or her ability to achieve these goals or realize these values. Alternatively, the counselor may ask how changing the health behavior would be related to these goals or values. The list of values and attributes can be tailored to the particular client population or the health behavior being addressed. For example, the list for adolescents may include values such as "being popular" or "being mature," whereas for an older population the list may include values related to independent living or maintaining youth or vitality. Alternatively, some practitioners obtain goals and values from clients using open ended questions rather than a list.

**Table 1:** List of values, attributes, and goals

Good Parent	Attractive
Good Spouse/Partner	Disciplined
Good Community Member	Responsible
Strong	In Control
On top of things	Respected at work
Competent	Athletic
Spiritual	Not hypocritical
Respected at home	Energetic
Good Christian (or Jew, Muslim etc)	Considerate
Successful	Youthful

In standard medical and health counseling practice, practitioners often provide information about the risks of continuing a behavior or the benefits of change with the intent of persuading the client. A traditional counseling statement might be, "It is very important that you change." In this style of highly directive counseling or unsolicited advice, the practitioner often attempts to instill motivation by increasing the client's perceived risk. This type of communication can elicit reactance, or push back from the client[56,57].

In contrast, in MI information is presented using an ELICIT-PROIVDE-ELICIT framework. The counselor first *elicits *the person's understanding and need for information, then *provides *new information in a neutral manner, followed by *eliciting *what this information might mean for client, using a question such as, "What does this mean to you" or "How do you make sense of all this?" MI practitioners avoid trying to persuade clients with "pre-digested" health messages and instead allow clients to process information and find what is personally relevant for them. Autonomy is supported by also asking how much information the client might desire.

##### Moving from why to how: a three phase model of motivational interviewing

A key challenge for many clinicians learning MI is determining when and how to transition from building motivation to planning a course of action. Once resistance or ambivalence are resolved and motivation is solidified, many practitioners struggle with how to transition the discussion to action planning while still retaining the spirit of client centeredness; moving from the WHY phase to the HOW phase in a style that is MI-consistent. For many, there is a perception that the counseling style, skills, and strategies used to build motivation are distinct from those used in action planning. This can lead to a fractured clinical experience for both the counselor and the client.

The WHY to HOW transition does not, however, necessitate abandoning a client-centered style for a more overtly educational or directive style. There have been prior attempts to unify the WHY and HOW components of MI and action therapies, such as CBT [58,59]. However, these prior efforts to combine MI and CBT have in effect, simply pasted the two components together, with MI serving as a prelude or pretreatment motivational primer. Less work has been done on truly integrating the two approaches. In particular there is a need to develop autonomy supportive variants of CBT and other action oriented approaches that are conceptually consistent with MI, not only stitched together. To this end, we propose a three-component model of MI comprising three core tasks: *Exploring, Guiding*, and *Choosing*. This model is an adaptation of models that Rollnick et al. [18,60] previously proposed. Each task or phase is characterized by different counseling objectives and usually applies specific skills and techniques.

#### Task 1: Exploring

The primary objective during this phase is to "comfort the afflicted." Counselors elicit the client's story, build rapport, obtain a behavioral history that includes prior attempts to change, and collaboratively with the client decide what behaviors to address during the session. Key skills used during this phase include listening, shared agenda setting, open-ended questions, content, feeling, and double-sided reflections. The counselor conveys empathy and demonstrates that s/he will not prematurely request that the client change. There is little action planning during this phase, although the counselor may "parking lot" ideas that start to emerge with a verbal note to revisit them later in the encounter.

#### Task 2: Guiding

Once rapport has been established and the essence of the client's story has been evoked, the discussion can move to Guiding. During this phase, the counselor may "afflict the comfortable" by moving the conversation toward the possibility of change. The counselor elicits change talk by asking the client to consider life with and without change and by building discrepancy between the client's current actions and his or her broader life goals and values. Key strategies used during this phase include 0-to-10 importance/confidence rulers, a values clarification exercise, and summarizing. Phase II typically concludes with the counselor summarizing what was discussed including potential reasons for making a change, and asking the client something along the lines of, "so where does that leave you?", or "given this, what small change might you be willing to undertake?". If the client expresses a clear commitment to making a change, even if small in magnitude, the session can move to Phase III and a more pragmatic discussion of HOW to implement said change.

#### Task 3: Choosing

This is where action oriented approaches are brought to bear. Primary objectives during this phase include helping clients identify a goal, building an action plan, anticipating barriers, and agreeing on a plan for monitoring and if applicable, contingencies for successful effort. Key skills used in this phase include menu building and goal setting, typically executed through the action reflection.

When building an action plan there is the potential for the client to refute suggestions, even if they are derived from their prior statements, and offered in a tentative, "undersold" tone by the clinician. They may elicit a "yes-but" response, either due to underlying resistance or simply because the option does not work for the client based on intuition or experience. It is important to bear in mind that, like any type of reflection, action reflections, represent the clinician's best guess for what the client said or where the story is going. They are hypotheses and the productive "foul tip" rule applies here as well. That is, action reflections that are rejected by the client can still yield productive information about what does and does work or what should or should not be pursued, which nonetheless helps move the conversation toward resolution. One means to minimize outright rejection of an action reflection is to provide multiple options within the reflection. For example, "based on what you have said, it appears you have a few options available....", or in the shortened form "x or y, might be helpful here". Choice reduces reactance.

Although the three-task model implies a temporal sequence of Explore-Guide-Choose, and this will often be the case for patients entering counseling in an ambivalent or resistant state, not all clients will follow this linear order. Some patients may come into counseling fully motivated to change and may benefit from Choosing earlier on, whereas others may require recycling through Exploring and Guiding before they can commit to change.

## Conclusion

As proposed by Vansteenkiste et al in this issue (Int J Behav Nutr Phys Act. 2012 Mar 2;9(1):19), MI and SDT developed along quite different pathways, with the former being driven more by practice and induction and the latter driven more by theory and experimental research. Despite their unique origins, a marriage between MI and SDT may be helpful for both clinicians and theoreticians. For MI clinicians, a more acute awareness of SDT's core needs of autonomy, competence, and relatedness can provide a theoretical framework to guide the format and content of their counseling. Conversely, for SDT theorists, MI can provide a well-established framework through which to apply their concepts clinically. The three-phase model of MI proposed herein can serve as such a unifying framework for MI clinicians to apply SDT concepts across the full spectrum of clinical practice.

Further, using SDT as the theoretical basis for MI, even if only de facto, can help guide research and practice in other ways. For example, how do MI and SDT handle individual and cultural difference in counseling style preferences. Although many patients report satisfaction with and improved outcomes from patient-centered approaches [61-63] such as MI, some individuals may prefer and benefit from a more directive counseling style[64]. In one recent study [65], where rural African American women viewed an MI training tape showing both MI and non-MI consistent practice, many expressed concern that the MI consultation was *too *patient centered, ''He [provider] was asking the patient more about his decision, instead of him [provider] telling him.'' Another patient stated, ''He [health care provider] [was] not giving the patient much information. He's supposed to know, he's a doctor.'' Many patients implied that a more practitioner-centered, directive approach, where the health care provider did most of the talking and offered unsolicited advice, was desired. In a recent study we just completed with Mexican Americans with diabetes a substantial proportion indicated that they just "wanted their doctor to tell them what to do." How do we address patient requests for a more directive style of counseling while at the same time respecting autonomy and encouraging volition? On one hand, SDT would assume a priori that autonomy is a universal need, and any counseling must support client autonomy. So then, how can "directive" counseling still be autonomy supportive? How can practitioners offer advice while still supporting volition? Given that some clients may respond better to directive advice, there is a need for methods to assess how open clients are to internalizing direct advice versus feeling controlled by and reactive to it. SDT might propose that even if the source of advice is external, if the person has requested such advice and the clinician still frames the discussion with volition, then autonomy is maintained. And although autonomy is one core need, sometimes, as in the case above, clinicians may need to focus more on relatedness and competence than autonomy. SDT might help MI clinicians think differently about what client needs to focus on during different phases of counseling and how to address different types of patients.
